# Weak and vanishing upper mantle discontinuities generated by large-scale lithospheric delamination in the Longmenshan area, China

**DOI:** 10.1038/s41598-021-01061-4

**Published:** 2021-11-03

**Authors:** Chuansong He

**Affiliations:** Institute of Geophysics, China Earthquake Administration, Beijing, 100081 China

**Keywords:** Solid Earth sciences, Physics

## Abstract

A large amount of high-quality teleseismic data is used for common conversion point (CCP) stacking of receiver functions in the Longmenshan area and near-by region. The results show that a large-scale high-velocity anomaly (LHVA) or lithospheric delamination can modify the structure of upper mantle discontinuities or weaken the phase boundary of olivine, which is a very important finding and can be used to assess stagnant slabs in the mantle transition zone globally. The deepening region of the 660 km discontinuity beneath the Songpan-Ganzi terrane identified in this study might be generated by the LHVA.

## Introduction

The Longmenshan region has undergone two orogenic events since the Mesozoic: east–west deformation induced by the Indosinian orogeny in the Late Triassic–Early Jurassic^1^ and south-north deformation linked to the assembly or collision between the Indian and Asian continents in the Cenozoic^2,3^. This assembly has subsequently led to large-scale crustal shortening across Asia and greatly deformed the Longmenshan area^4–7^.

GPS studies have reported that crustal materials move eastward from the Tibetan Plateau into the Longmenshan area in the Cenozoic^8^, but they are obstructed by the rigid crust and lithosphere of the Yangtze Block or the Sichuan Basin^9,10^. This might result in stress accumulation and release in this area^11^ and lead to several devastating earthquakes in the Longmanshan area, such as the 2001 Mw 7.8 Kunlun, the 2008 Mw 7.9 Wenchuan, the 2010 Mw 6.9 Yushu and the 2013 Ms 7.0 Lushan earthquakes.

A previous tomographic study suggested that the assembly and collision among the North China, Yangtze and Qiangtang terranes during the Indosinian period in the Mesozoic or the northward subduction and amalgamation of the Indian plate during the Cenozoic resulted in delamination of the lithospheric structure in the Longmenshan area in the mantle transition zone (MTZ)^11^_,_ which might facilitate the eastern extrusion of the Tibetan Plateau^12^ as well as ductile crustal thickening^13,14^. However, other tomography techniques did not define such LHVA or delamination bodies in this area^15,16^. Therefore, it is necessary to further investigate and confirm this deep process.

**Figure 1:**
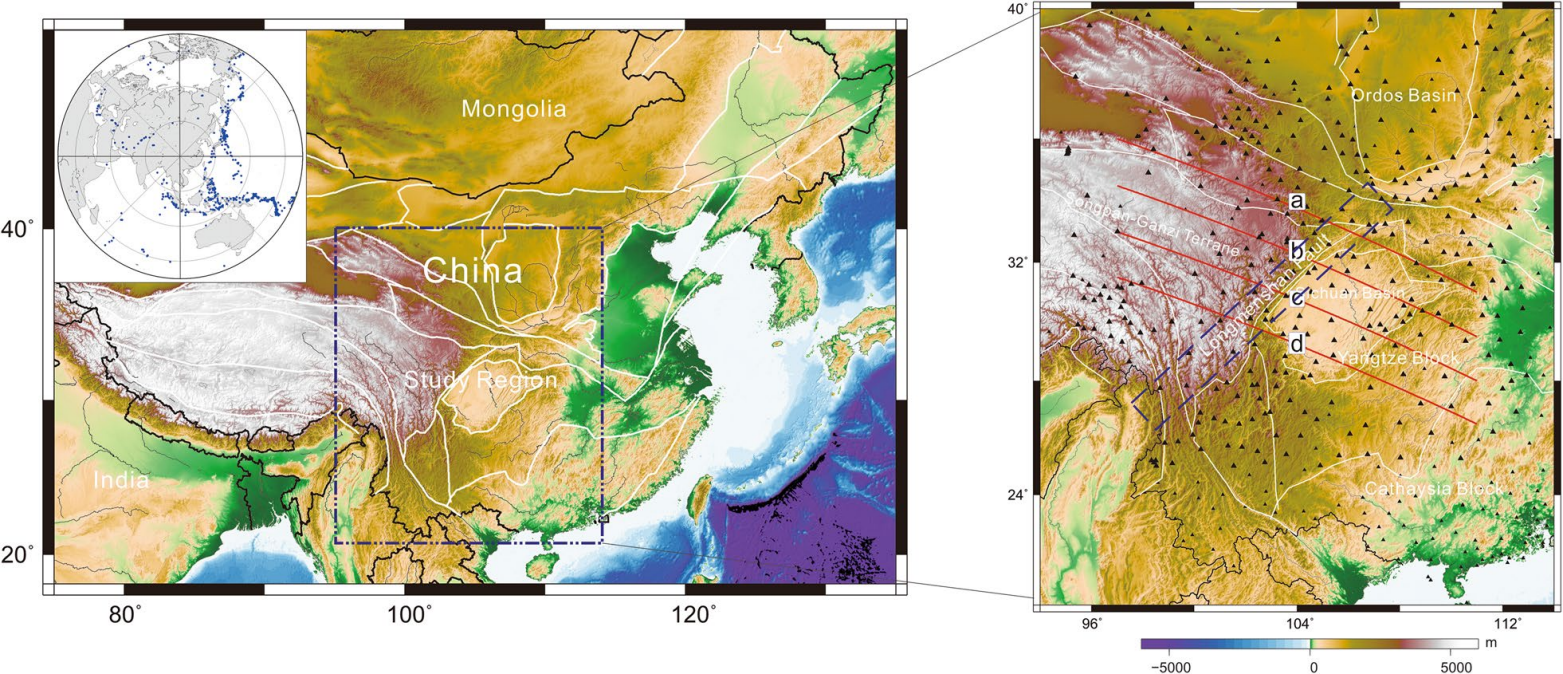
Left panel: location of the study region; inset figure: distribution of events used in this study. Right panel: distribution of seismic stations and tectonic framework; blue rectangle is the Longmenshan Orogen; white lines: boundaries of geological units; red lines: profiles for CCP stacking of receiver functions; profiles b and c also show P-wave velocity perturbations^11^ [the figure was generated by Chuansong He using the Generic Mapping Tool (http://gmt.soest.hawaii.edu/)].

Generally, a high-velocity anomaly (or delamination body) is a cold domain, which might induce structural variations in the MTZ^17^. The receiver function technique is an effective tool that can be used to detect the structure of the MTZ. Accordingly, I collected a large amount of high-quality teleseismic data (Fig. 1, left panel), carried out common conversion point (CCP) stacking of receiver functions and imaged the structure of the MTZ. The results show that the LHVA can result in weak or modifying upper mantle discontinuities, which also demonstrate that the lithosphere has delaminated into the MTZ.

## Results and discussion

**Figure 2:**
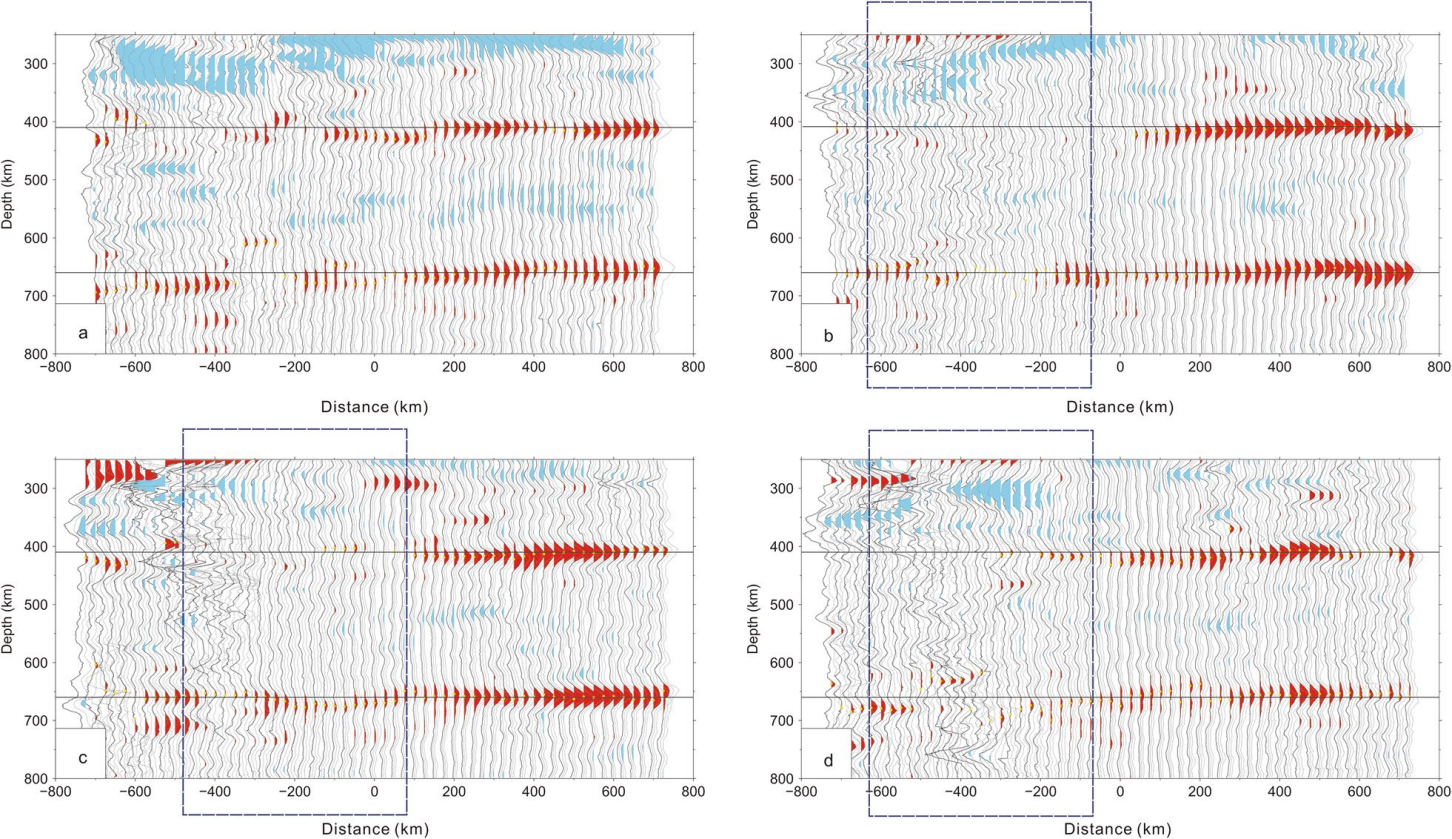
CCP stacking profiles of receiver functions (**a**–**d**) corrected by a 3-D global P- and S-wave velocity model^18^. Blue rectangular region: weak and vanishing 410 km and 660 km discontinuities. The yellow points are selected for the depths of both the 410 km and 660 km discontinuities on the CCP stacking of receiver functions (Supplementary Tables S1, S2, S3 and S4). The bootstrapping method with 2000 stacked amplitudes is used to resample and calculate the dataset, and the final mean receiver functions corresponding to the 95% confidence level are calculated. Horizontal blue lines: depths of 410 and 660 km [the figure was generated by Chuansong He using the Generic Mapping Tool (http://gmt.soest.hawaii.edu/)].

Four CCP stacking profiles of receiver functions, which are corrected by a global P- and S-wave velocity model^18^, were obtained (Fig. 2; for the locations of the profiles, see Fig. 1, right panel). The results show that the amplitude of the 410 km discontinuity becomes small in the western part of profile a, whereas the 410 km discontinuity almost vanishes in the western parts of profiles b, c and d. Moreover, the local 660 km discontinuity also weakens in the western parts of profiles b, c and d.

**Figure 3:**
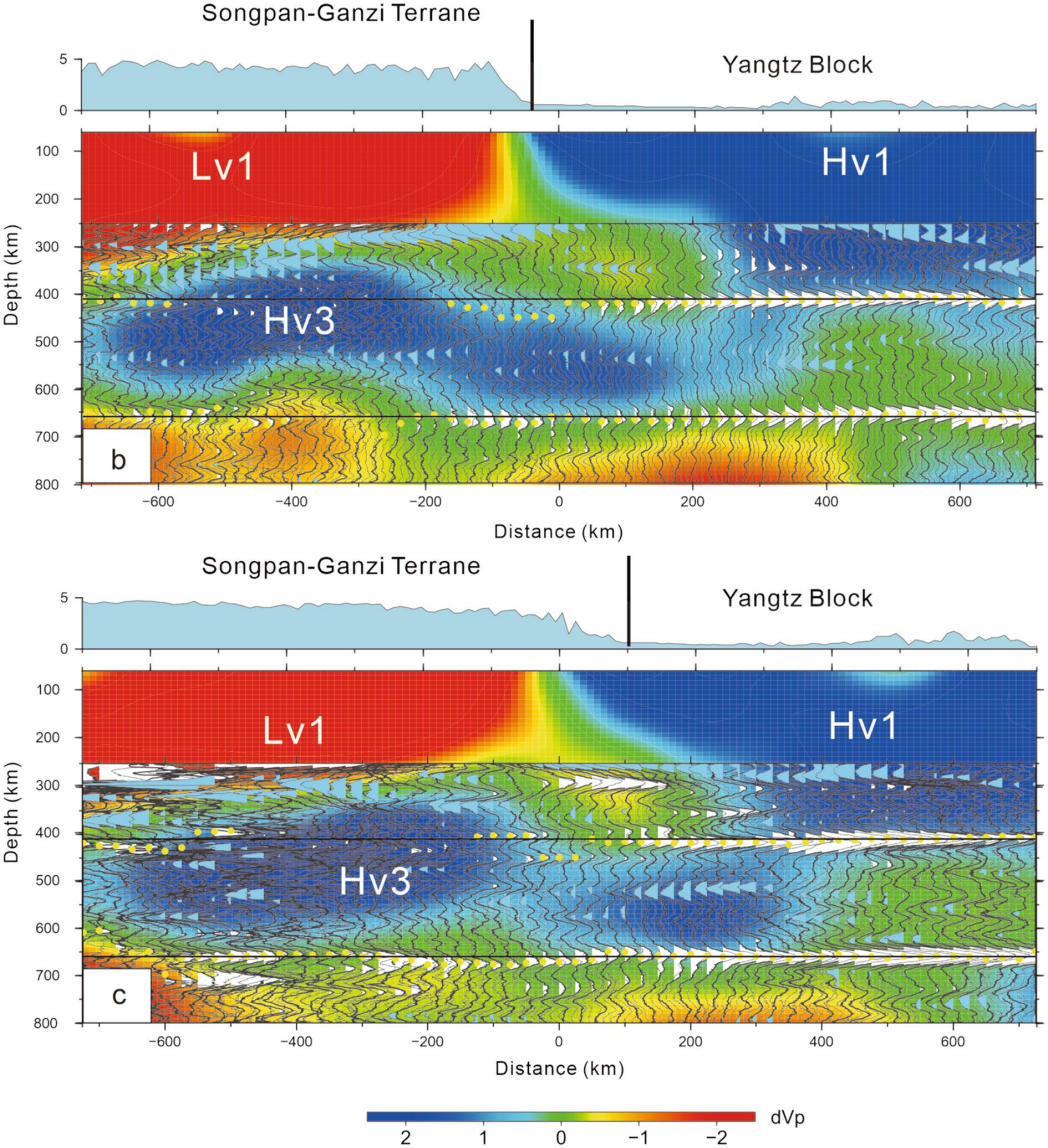
Profiles (**b**) and (**c**). Overlapping diagram of the P-wave velocity perturbation^18^ and the CCP stacking profiles (location of profiles (c) and (d); see the locations in Fig. 1). A weak or vanishing 410 km discontinuity corresponds to the LHVA (Hv3) or large-scale lithospheric delamination. The yellow points are selected for the depths of both the 410 km and 660 km discontinuities in the CCP stacking of receiver functions. The bootstrapping method with 2000 stacked amplitudes is used to resample and calculate the dataset, and the final mean receiver functions corresponding to the 95% confidence level are calculated. Horizontal blue lines: depths of 410 and 660 km [the figure was generated by Chuansong He using the Generic Mapping Tool (http://gmt.soest.hawaii.edu/)].

**Figure 4:**
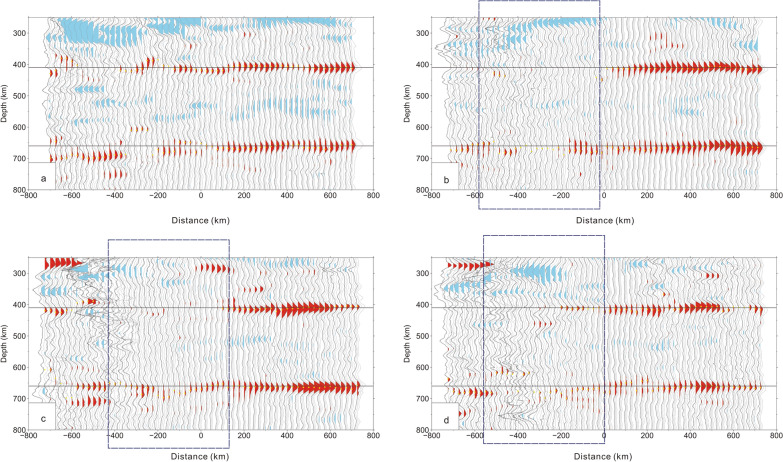
CCP stacking profiles of receiver functions (**a**–**d**) corrected by a local velocity model^19^. Blue rectangular region: weak and vanishing 410 km and 660 km discontinuities. The yellow points are selected for the depths of both the 410 km and 660 km discontinuities on the CCP stacking of receiver functions. The bootstrapping method with 2000 stacked amplitudes is used to resample and calculate the dataset, and the final mean receiver functions corresponding to the 95% confidence level are calculated. Horizontal blue lines: depths of 410 and 660 km [the figure was generated by Chuansong He using the Generic Mapping Tool (http://gmt.soest.hawaii.edu/)].

To further check this issue, P-wave velocity perturbation profiles^11^ overlap with the CCP stacking profiles (b and c) (Fig. 3). The results indicate that the vanishing or weak 410 km discontinuity in the western parts of profiles b and c corresponds well to the high-velocity anomaly (Hv3)^11^. Clearly, the LHVA (Hv3) modified the 410 km discontinuity or made the phase boundary of olivine vanish. To date, no such case has been reported anywhere in the world. A local velocity model is also adopted to remove the velocity heterogeneity effect in the upper mantle^19^. The CCP stacking results of the receiver functions indicate that the results are almost consistent with the above results (Fig. 4).

**Figure 5:**
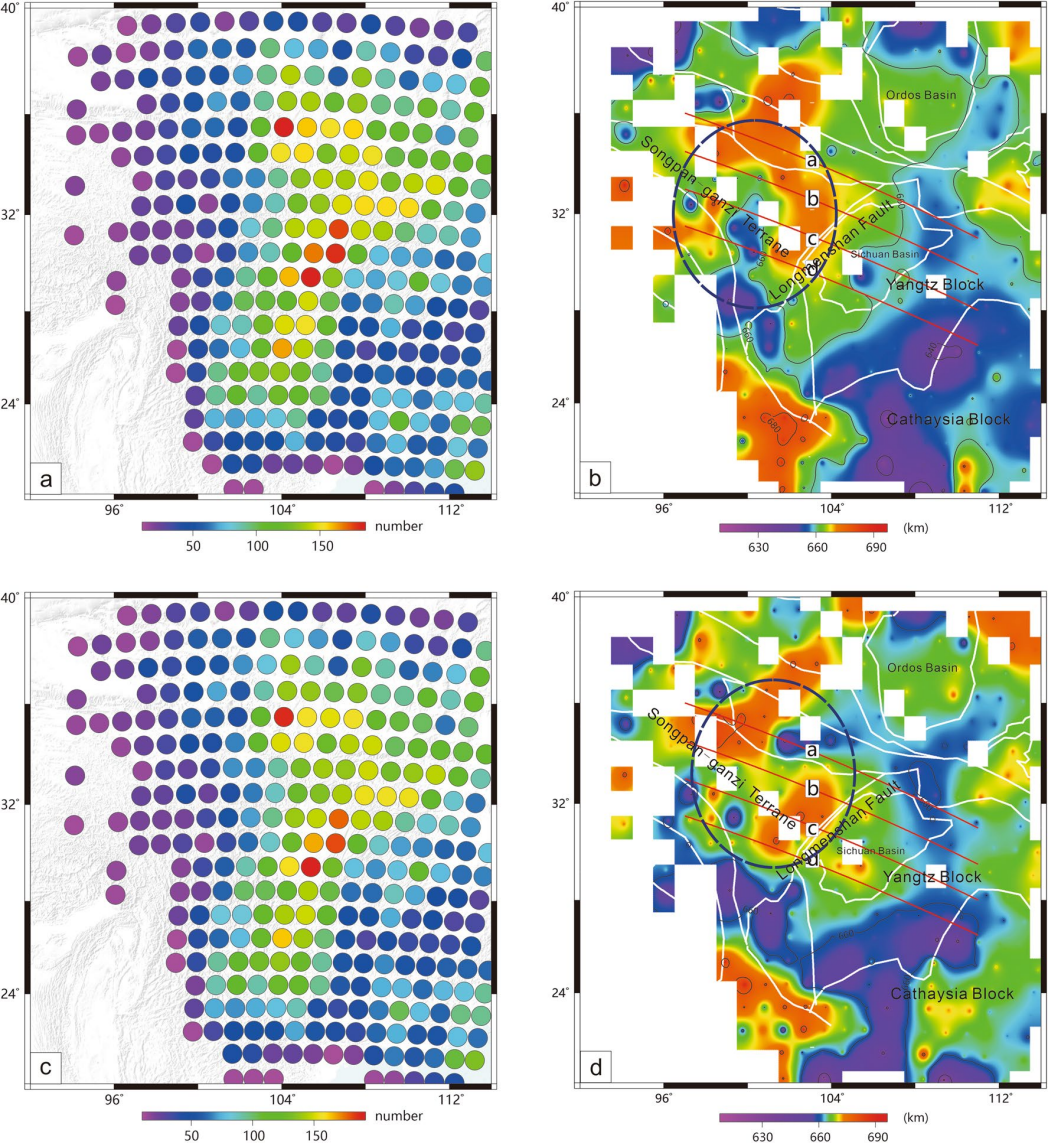
CCP stacking points of receiver functions, which are corrected by a 3-D global P- and S-wave velocity model (**a**,**b**)^18^ and corrected by a 3-D local velocity model (**c**,**d**)^19^. A stacking point with more than 10 points can be used to calculate the depth of the 660 km discontinuity (**a**,**c**). Topography of the 660 km discontinuity (**b**,**d**), blue elliptical region: the deepening region of the 660 km discontinuity. Red lines: CCP stacking profiles (please see Figs. 2 and 4) [the figure was generated by Chuansong He using the Generic Mapping Tool (http://gmt.soest.hawaii.edu/)].

I have attempted to extract the topographies of the 410 km and 660 km discontinuities. However, due to the weak and vanishing 410 km discontinuity in the western part of the study region, obtaining the complete topography of the 410 discontinuity is very difficult. Accordingly, I extract the topography of only the 660 km discontinuity in this area (Fig. 5). The results show that the 660 km discontinuity beneath the Songpan-Ganzi terrane is deepened by an average of approximately 20–30 km (Fig. 5b,d, blue elliptical region) compared to the global topography of the 660 km discontinuity^20–22^, which correspond well to the distribution of the LHVA (Supplementary Fig. S1, 400 km depth section, Hv3)^11^.

Experimental studies have revealed that upper mantle discontinuities might be associated with phase changes in olivine^23,24^. The 410 km discontinuity has a positive pressure–temperature slope that involves the olivine-to-wadsleyite transition^25^. The phase change of the 660 km discontinuity in the garnet system is thought to be dominant in the relatively high temperature situation with a positive pressure–temperature gradient. The depth of the 660 km discontinuity will be deepened when the temperature increase in the garnet system, whereas phase changes of the 660 km discontinuity in the olivine system are suggested to be dominant in the relatively low-temperature situation^26^, which exhibits a negative pressure–temperature gradient that involves the ringwoodite phase transformation to perovskite and magnesiowüstite^27,28^. When the temperature increases, the depth of the 660 km discontinuity involved with the olivine system will become shallow, whereas when the temperature decreases, the depth of the 660 km discontinuity will become deepening.

A high-velocity anomaly, such as a slab, is generally considered a lower-temperature domain than the surrounding mantle^17,29^; thus, inferring that the deepening topography of the 660 km discontinuity beneath the Songpan-Ganzi terrane might be associated with the LHVA (Supplementary Fig. S1, Hv3).

The situation of the LHVA (Fig. 3) is very similar to that of a stagnant slab in the MTZ. Therefore, the effect of lithospheric delamination in the MTZ can be used analogously to the stagnant slab in the MTZ. In other words, the vanishing or weak topography of upper mantle discontinuities may be used as a standard to test the existence of the stagnant slab in the MZT.

## Conclusions

1. Weak or vanishing upper mantle discontinuities correspond well to the LHVA, which might indicate or demonstrate that the lithosphere has delaminated into the MTZ.
2. The LHVA or lithospheric delamination can modify the structure of upper mantle discontinuities or make them weak (or vanishing), which is first discovered in the world.
3. The situation of the LHVA beneath the Songpan-Ganzi terrane is very similar to that of a stagnant slab in the MTZ. Therefore, this result may be used to understand or assess a stagnant slab in the MTZ, which is an issue that generates great controversy. In particular, it can be used to test whether the stagnant slab in the MTZ was generated by the western subduction of the Pacific Plate beneath Eastern China or Eastern Asia.

## Data and methods

In total, 684 teleseismic events were collected from 406 permanent seismic stations recorded during 2007–2020 (Fig. 1), with earthquake epicentral distances ranging from 30° to 90° for individual event-station pairs and Ms > 6.0 (Fig. 1, insert in left panel). The raw record was cut from 15 s before to 200 s after the P-wave arrival and filtered by a Butterworth bandpass filter between 0.01 and 0.2 Hz. To select consistent raw data for the waveforms, a cross-correlation technique^30^ was used for data processing (for example, see Supplementary Fig. S2). Finally, 16,258 high-quality receiver functions were extracted by a modified frequency-domain deconvolution (0.01 water level and 1 Gaussian factor)^31,32^ (for example, see Supplementary Fig. S3).

The technique of CCP stacking of receiver functions is employed to define the topographies of the 410 and 660 km discontinuities^33–35^, and spherical coordinates are established to calculate the Ps-P differential time *Tp*_*s*_^33^:

1 $${T}_{Ps}=\sum_{i}^{N}\left(\sqrt{\left(\frac{{R}_{i}}{{V}_{Si}}\right)-{p}_{{P}_{s}}^{2}}-\sqrt{\left(\frac{{R}_{i}}{{V}_{Pi}}\right)-{p}_{P}^{2}}\right)\frac{\Delta r}{{R}_{i}},$$

where the ray parameters of the direct Ps and P phases are represented as p_Ps_ and p_P_, respectively. V_Pi_ and V_Si_ are the P- and S-wave velocities in the *i*th layer, and ${R}_{i}\mathrm{ and }\Delta \mathrm{r}$ represents the Earth’s semidiameter at each *i*th depth shell (${r}_{i}$) and depth interval. A 3-D global P- and S-wave velocity model by Lu et al.^17^ is used to remove the velocity heterogeneity effects in the upper mantle, and a 3-D local velocity model^18^ is also used to remove the effects of velocity heterogeneities in the upper mantle and to estimate the S-wave velocity based on the Vp/Vs ratio of the AK135 velocity model^36^. The Ps-P differential times in the 3-D model are presented as follows:

2 $${T}_{Ps3D}={T}_{Ps}+\Delta T,$$

where $\Delta T$ is related to the travel-time correction or the 3-D velocity perturbations. The high- and low-velocity anomalies in the upper mantle can result in a travel-time increase or decrease (*ΔT*) of a ray and lead to deviations in the real depths of the 410 and 660 km discontinuities. In the CCP stacking of receiver functions, the lateral grid interval and depth interval are designed as 0.5° and 1 km, respectively, and the search radius (or bin) of the migrated receiver functions is designated as 75 km^35^. In each bin, bootstrap resampling with 2000 resampling iterations^37^ is used to calculate the mean value and standard deviation. Piercing points are calculated by the 1-D AK135 velocity model^36^, which shows good and reasonable piercing point distributions at depths of 410 and 660 km (Supplementary Fig. S4).

## Supplementary Information

Supplementary Information.

## Data Availability

The raw data used for CCP stacking of receiver functions can be accessed at https://doi.org/10.5281/zenodo.5035828.
