# Supplementary material for: Weak and vanishing upper mantle discontinuities generated by large-scale lithospheric delamination in the Longmenshan area, China
Source: Sci Rep. 2021 Nov 3;11:21580. doi: 10.1038/s41598-021-01061-4 (PMC8566558; doi:10.1038/s41598-021-01061-4)
Supplement: Supplementary file 1 — Supplementary Information. [file 41598_2021_1061_MOESM1_ESM.docx]

**Weak and vanishing upper mantle discontinuity generated by a large-scale lithospheric delamination in the Longmenshan area, China**

Chuansong He

Institute of geophysics, China Earthquake Administration, 100081, Beijing

Corresponding author: Chuansong He, email: hechuansong@aliyun.com


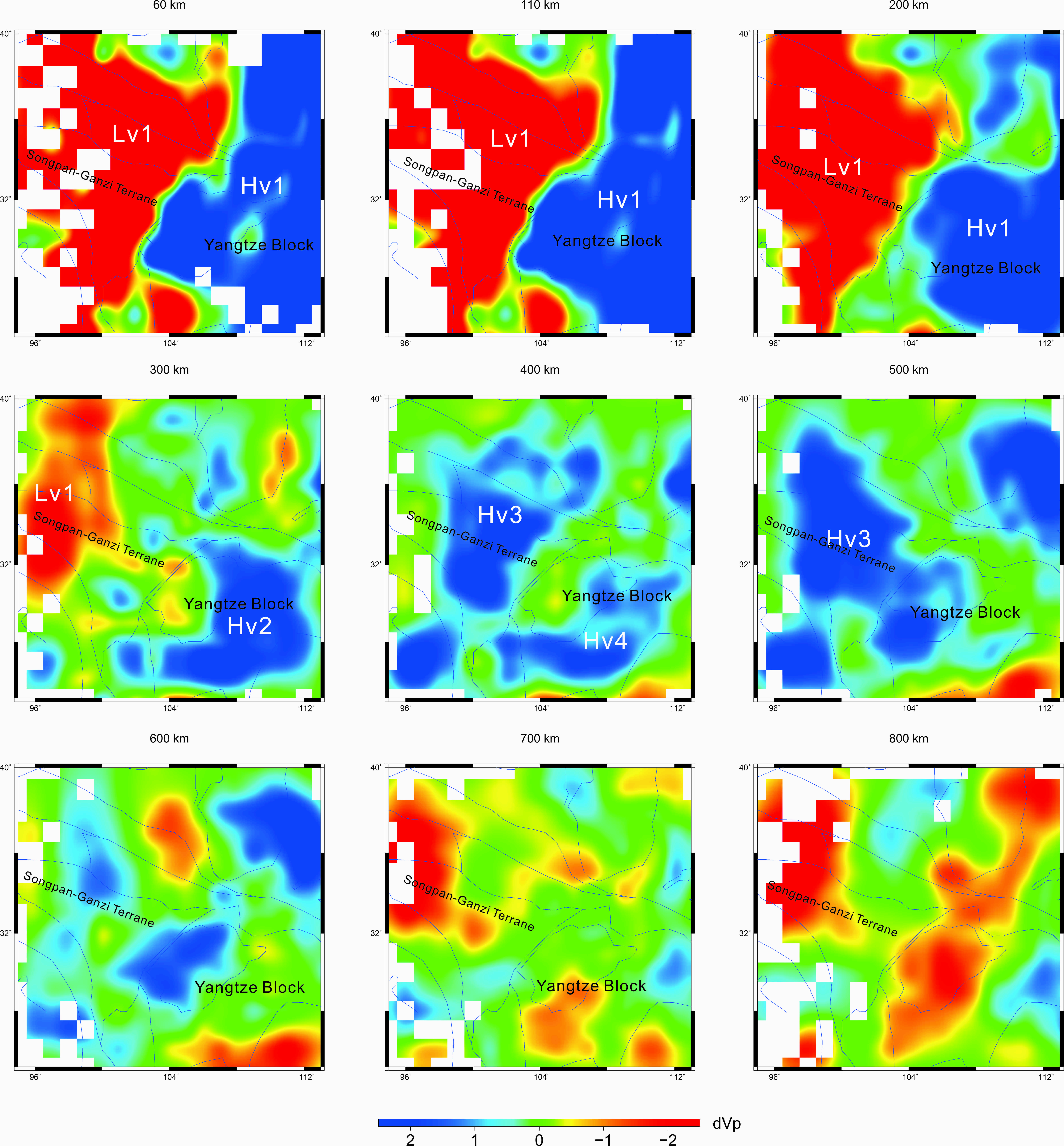


Fig. S1. P-wave perturbation at depths of 50-800 km ^11^ the figure was generated by Chuansong He using the Generic Mapping Tool (http://gmt.soest.hawaii.edu/)).


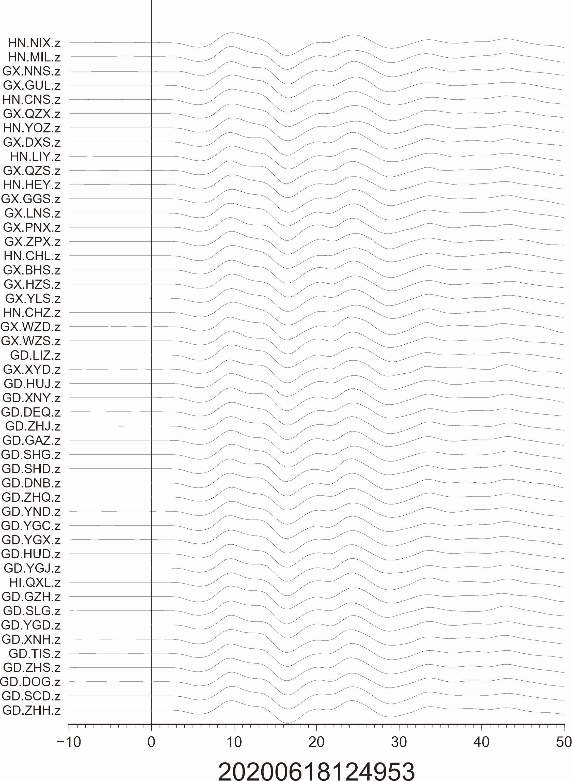


Fig. S2. Event: 20200618124953. Horizontal coordinate: second, vertical coordinate: vertical component of seismic stations (the figure was generated by Chuansong He using the Generic Mapping Tool (http://gmt.soest.hawaii.edu/)).


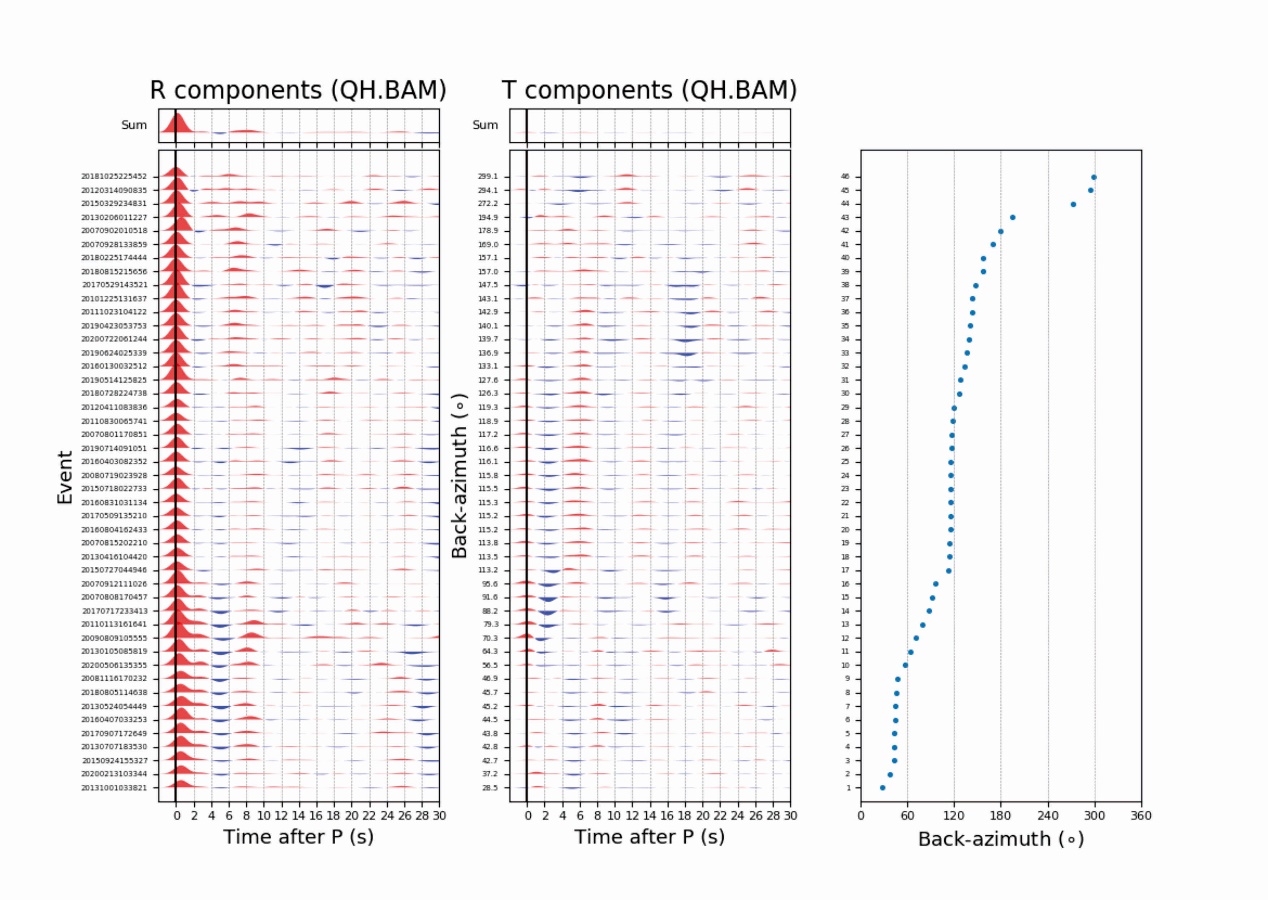


Fig. S3. R and T receiver functions of QH.BAM station (the figure was generated by Chuansong He using the Generic Mapping Tool (http://gmt.soest.hawaii.edu/)).


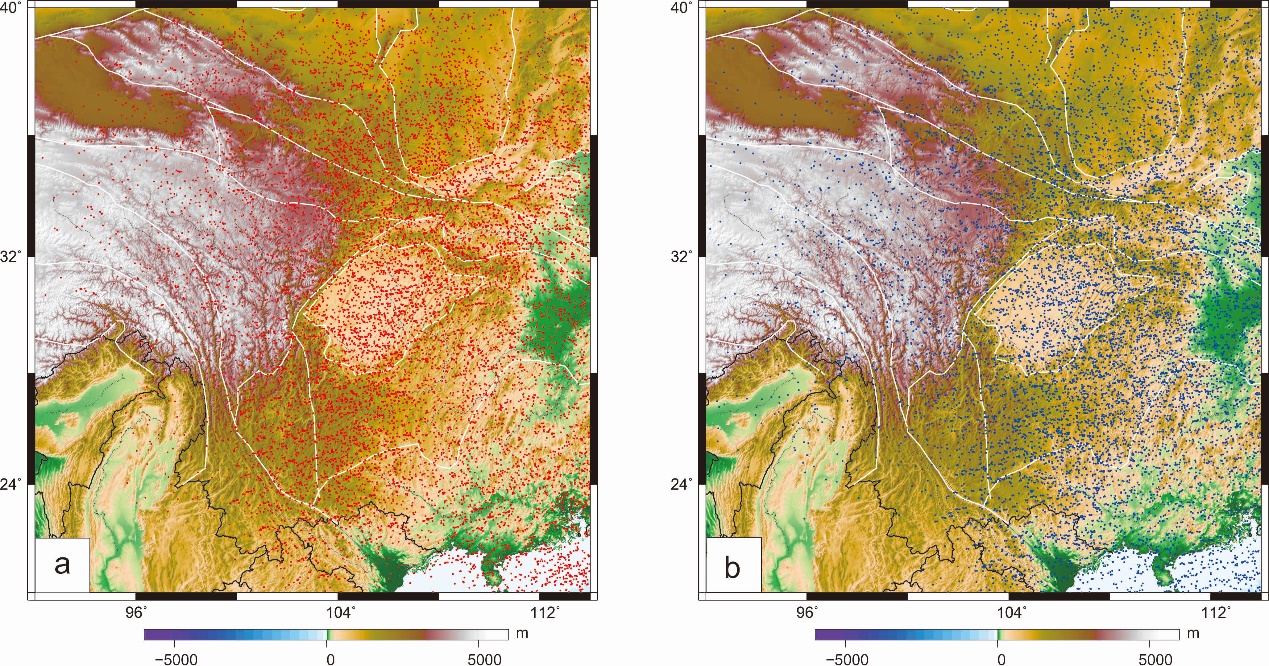


Fig. S4. Piercing points (red points) at the depth of the 410 km (a) and piercing points (blue point) at the depth of 660 km (b) (the figure was generated by Chuansong He using the Generic Mapping Tool (http://gmt.soest.hawaii.edu/)).

Table S1: profile a

| Distance (km) | 660 km discontinuity | Peak amplitude of the 660 km discontinuity | Stacking points | 410 km discontinuity | Peak amplitude of the 410 km discontinuity | Stacking points |
| --- | --- | --- | --- | --- | --- | --- |
| -700 | 694 | 0.03696 | 25.0 |  |  |  |
| -675 | 693 | 0.02877 | 29.0 |  |  |  |
| -650 | 691 | 0.02650 | 27.0 |  |  |  |
| -625 | 686 | 0.02258 | 24.0 |  |  |  |
| -600 | 679 | 0.02131 | 24.0 |  |  |  |
| -575 | 681 | 0.02325 | 31.0 |  |  |  |
| -550 | 686 | 0.02881 | 34.0 |  |  |  |
| -525 | 686 | 0.02773 | 41.0 |  |  |  |
| -500 | 689 | 0.02885 | 51.0 |  |  |  |
| -475 | 685 | 0.03089 | 60.0 |  |  |  |
| -450 | 685 | 0.03039 | 65.0 |  |  |  |
| -425 | 677 | 0.03254 | 60.0 |  |  |  |
| -400 | 678 | 0.03163 | 60.0 |  |  |  |
| -375 | 678 | 0.03086 | 49.0 |  |  |  |
| -350 | 677 | 0.01865 | 44.0 |  |  |  |
| -325 | 610 | 0.02130 | 42.0 |  |  |  |
| -300 | 610 | 0.01981 | 49.0 |  |  |  |
| -275 | 606 | 0.01928 | 53.0 |  |  |  |
| -250 | 610 | 0.01678 | 55.0 |  |  |  |
| -225 | 666 | 0.01391 | 55.0 |  |  |  |
| -200 | 669 | 0.01906 | 66.0 |  |  |  |
| -175 | 678 | 0.02282 | 79.0 |  |  |  |
| -150 | 678 | 0.02180 | 93.0 |  |  |  |
| -125 | 678 | 0.02029 | 101.0 |  |  |  |
| -100 | 648 | 0.02031 | 113.0 |  |  |  |
| -75 | 648 | 0.02093 | 112.0 | 419 | 0.02958 | 95.0 |
| -50 | 680 | 0.01697 | 117.0 | 421 | 0.02724 | 99.0 |
| -25 | 678 | 0.01912 | 126.0 | 423 | 0.02161 | 119.0 |
| 0 | 678 | 0.01639 | 117.0 | 423 | 0.02359 | 127.0 |
| 25 | 669 | 0.01431 | 135.0 | 427 | 0.02727 | 149.0 |
| 50 | 661 | 0.01517 | 116.0 | 430 | 0.02914 | 132.0 |
| 75 | 671 | 0.02270 | 127.0 | 430 | 0.02979 | 126.0 |
| 100 | 666 | 0.02236 | 144.0 | 425 | 0.02836 | 126.0 |
| 125 | 668 | 0.02841 | 146.0 | 425 | 0.02067 | 139.0 |
| 150 | 669 | 0.02754 | 137.0 | 409 | 0.02107 | 157.0 |
| 175 | 669 | 0.02610 | 141.0 | 409 | 0.02569 | 174.0 |
| 200 | 663 | 0.02653 | 148.0 | 411 | 0.03191 | 176.0 |
| 225 | 670 | 0.02687 | 142.0 | 410 | 0.03547 | 156.0 |
| 250 | 659 | 0.02194 | 148.0 | 410 | 0.03285 | 159.0 |
| 275 | 658 | 0.02707 | 141.0 | 410 | 0.03642 | 144.0 |
| 300 | 659 | 0.03053 | 154.0 | 411 | 0.03708 | 145.0 |
| 325 | 662 | 0.03013 | 164.0 | 409 | 0.03412 | 137.0 |
| 350 | 658 | 0.02863 | 145.0 | 409 | 0.02811 | 148.0 |
| 375 | 657 | 0.02744 | 142.0 | 408 | 0.02521 | 138.0 |
| 400 | 653 | 0.02088 | 159.0 | 410 | 0.02126 | 153.0 |
| 425 | 652 | 0.02090 | 140.0 | 409 | 0.01897 | 133.0 |
| 450 | 652 | 0.02328 | 137.0 | 414 | 0.01596 | 107.0 |
| 475 | 654 | 0.02412 | 154.0 | 415 | 0.01810 | 89.0 |
| 500 | 662 | 0.02439 | 159.0 | 415 | 0.02761 | 84.0 |
| 525 | 661 | 0.02777 | 156.0 | 415 | 0.04163 | 83.0 |
| 550 | 664 | 0.03071 | 131.0 | 418 | 0.03888 | 85.0 |
| 575 | 664 | 0.02621 | 117.0 | 416 | 0.04021 | 89.0 |
| 600 | 656 | 0.03001 | 110.0 | 415 | 0.03841 | 100.0 |
| 625 | 660 | 0.03081 | 95.0 | 414 | 0.04013 | 104.0 |
| 650 | 659 | 0.02573 | 93.0 | 412 | 0.03952 | 109.0 |
| 675 | 649 | 0.02930 | 84.0 | 412 | 0.03636 | 92.0 |
| 700 | 653 | 0.03643 | 69.0 | 414 | 0.03214 | 89.0 |

Table S2: profile b

| Distance (km) | 660 km discontinuity | Peak amplitude of the 660 km discontinuity | Stacking points | 410 km discontinuity | Peak amplitude of the 410 km discontinuity | Stacking points |
| --- | --- | --- | --- | --- | --- | --- |
| -712 | 660 | 0.02393 | 22.0 |  |  |  |
| -687 | 662 | 0.02437 | 26.0 |  |  |  |
| -662 | 664 | 0.02742 | 27.0 |  |  |  |
| -637 | 666 | 0.03069 | 29.0 |  |  |  |
| -612 | 649 | 0.02243 | 34.0 |  |  |  |
| -587 | 653 | 0.02273 | 37.0 |  |  |  |
| -562 | 651 | 0.03161 | 41.0 |  |  |  |
| -537 | 648 | 0.03442 | 36.0 |  |  |  |
| -512 | 641 | 0.02489 | 27.0 |  |  |  |
| -487 | 669 | 0.02099 | 27.0 |  |  |  |
| -462 | 675 | 0.03532 | 30.0 |  |  |  |
| -437 | 676 | 0.02890 | 37.0 |  |  |  |
| -412 | 669 | 0.02953 | 31.0 |  |  |  |
| -387 | 660 | 0.02056 | 34.0 |  |  |  |
| -362 | 651 | 0.02025 | 34.0 |  |  |  |
| -337 | 657 | 0.01698 | 37.0 |  |  |  |
| -312 | 657 | 0.01526 | 49.0 |  |  |  |
| -287 | 661 | 0.01362 | 53.0 |  |  |  |
| -262 | 698 | 0.01280 | 60.0 |  |  |  |
| -237 | 674 | 0.01348 | 57.0 |  |  |  |
| -212 | 658 | 0.02345 | 56.0 |  |  |  |
| -187 | 656 | 0.01808 | 64.0 |  |  |  |
| -162 | 662 | 0.02381 | 63.0 |  |  |  |
| -137 | 671 | 0.03068 | 57.0 |  |  |  |
| -112 | 675 | 0.02918 | 64.0 |  |  |  |
| -87 | 662 | 0.02929 | 70.0 |  |  |  |
| -62 | 674 | 0.02667 | 81.0 |  |  |  |
| -37 | 673 | 0.02301 | 90.0 |  |  |  |
| -12 | 659 | 0.01819 | 95.0 | 449 | 0.01399 | 120.0 |
| 13 | 661 | 0.01746 | 118.0 | 417 | 0.01485 | 148.0 |
| 38 | 664 | 0.01900 | 126.0 | 422 | 0.01925 | 146.0 |
| 63 | 671 | 0.02108 | 137.0 | 418 | 0.02424 | 152.0 |
| 88 | 665 | 0.02292 | 137.0 | 417 | 0.01998 | 183.0 |
| 113 | 664 | 0.02120 | 146.0 | 420 | 0.02392 | 193.0 |
| 138 | 669 | 0.02203 | 137.0 | 416 | 0.02884 | 178.0 |
| 163 | 666 | 0.02170 | 126.0 | 415 | 0.03189 | 186.0 |
| 188 | 662 | 0.02123 | 149.0 | 419 | 0.03048 | 184.0 |
| 213 | 664 | 0.02201 | 167.0 | 415 | 0.03746 | 142.0 |
| 238 | 661 | 0.02429 | 184.0 | 416 | 0.04294 | 140.0 |
| 263 | 663 | 0.02447 | 183.0 | 414 | 0.04012 | 141.0 |
| 288 | 661 | 0.03025 | 180.0 | 411 | 0.04426 | 133.0 |
| 313 | 662 | 0.03140 | 171.0 | 412 | 0.04161 | 142.0 |
| 338 | 656 | 0.03301 | 141.0 | 410 | 0.04128 | 129.0 |
| 363 | 656 | 0.03177 | 142.0 | 413 | 0.04937 | 98.0 |
| 388 | 656 | 0.03461 | 152.0 | 411 | 0.04981 | 95.0 |
| 413 | 653 | 0.03250 | 145.0 | 411 | 0.04633 | 90.0 |
| 438 | 651 | 0.03433 | 123.0 | 413 | 0.04999 | 87.0 |
| 463 | 653 | 0.03322 | 103.0 | 410 | 0.04899 | 83.0 |
| 488 | 654 | 0.04660 | 90.0 | 407 | 0.04913 | 89.0 |
| 513 | 653 | 0.05286 | 74.0 | 404 | 0.05416 | 98.0 |
| 538 | 652 | 0.04945 | 69.0 | 405 | 0.05475 | 103.0 |
| 563 | 654 | 0.05117 | 74.0 | 409 | 0.05056 | 95.0 |
| 588 | 656 | 0.04644 | 78.0 | 408 | 0.04946 | 89.0 |
| 613 | 664 | 0.04181 | 86.0 | 409 | 0.03877 | 94.0 |
| 638 | 668 | 0.03974 | 96.0 | 415 | 0.03528 | 72.0 |
| 663 | 659 | 0.04969 | 102.0 | 417 | 0.05406 | 77.0 |
| 688 | 660 | 0.05264 | 103.0 | 414 | 0.05063 | 70.0 |
| 713 | 657 | 0.04795 | 102.0 | 416 | 0.05145 | 65.0 |

Table S3: profile c

| Distance (km) | 660 km discontinuity | Peak amplitude of the 660 km discontinuity | Stacking points | 410 km discontinuity | Peak amplitude of the 410 km discontinuity | Stacking points |
| --- | --- | --- | --- | --- | --- | --- |
| -725 | 608 | 0.02487 | 7.0 |  |  |  |
| -700 | 604 | 0.02672 | 19.0 |  |  |  |
| -675 | 649 | 0.02458 | 20.0 |  |  |  |
| -650 | 652 | 0.02374 | 18.0 |  |  |  |
| -625 | 649 | 0.01981 | 23.0 |  |  |  |
| -600 | 697 | 0.02389 | 24.0 |  |  |  |
| -575 | 663 | 0.02949 | 39.0 |  |  |  |
| -550 | 660 | 0.03033 | 46.0 |  |  |  |
| -525 | 663 | 0.03316 | 42.0 |  |  |  |
| -500 | 660 | 0.03373 | 47.0 |  |  |  |
| -475 | 658 | 0.03348 | 41.0 |  |  |  |
| -450 | 657 | 0.02702 | 30.0 |  |  |  |
| -425 | 653 | 0.02456 | 19.0 |  |  |  |
| -400 | 655 | 0.02332 | 25.0 |  |  |  |
| -375 | 656 | 0.02264 | 27.0 |  |  |  |
| -350 | 656 | 0.03047 | 29.0 |  |  |  |
| -325 | 651 | 0.03034 | 30.0 |  |  |  |
| -300 | 660 | 0.03008 | 40.0 |  |  |  |
| -275 | 658 | 0.03615 | 44.0 |  |  |  |
| -250 | 664 | 0.03069 | 50.0 |  |  |  |
| -225 | 667 | 0.02972 | 59.0 |  |  |  |
| -200 | 676 | 0.02391 | 72.0 |  |  |  |
| -175 | 677 | 0.02411 | 86.0 |  |  |  |
| -150 | 671 | 0.02227 | 71.0 |  |  |  |
| -125 | 673 | 0.02238 | 71.0 |  |  |  |
| -100 | 675 | 0.01734 | 89.0 |  |  |  |
| -75 | 676 | 0.01941 | 93.0 |  |  |  |
| -50 | 673 | 0.02236 | 95.0 |  |  |  |
| -25 | 671 | 0.02287 | 101.0 |  |  |  |
| 0 | 670 | 0.02191 | 112.0 | 449 | 0.01046 | 147.0 |
| 25 | 670 | 0.01968 | 118.0 | 449 | 0.01121 | 159.0 |
| 50 | 666 | 0.02192 | 127.0 | 410 | 0.01242 | 168.0 |
| 75 | 649 | 0.02003 | 140.0 | 417 | 0.01232 | 178.0 |
| 100 | 650 | 0.02110 | 143.0 | 415 | 0.01834 | 204.0 |
| 125 | 661 | 0.02064 | 151.0 | 416 | 0.02390 | 170.0 |
| 150 | 658 | 0.02121 | 172.0 | 417 | 0.02716 | 148.0 |
| 175 | 658 | 0.02292 | 182.0 | 419 | 0.02907 | 135.0 |
| 200 | 654 | 0.03048 | 161.0 | 419 | 0.03072 | 94.0 |
| 225 | 658 | 0.03516 | 150.0 | 417 | 0.04129 | 92.0 |
| 250 | 659 | 0.04100 | 133.0 | 418 | 0.04549 | 75.0 |
| 275 | 658 | 0.03692 | 122.0 | 416 | 0.04830 | 57.0 |
| 300 | 660 | 0.04130 | 93.0 | 416 | 0.04962 | 49.0 |
| 325 | 662 | 0.03902 | 81.0 | 415 | 0.03895 | 71.0 |
| 350 | 663 | 0.03017 | 78.0 | 422 | 0.04452 | 76.0 |
| 375 | 658 | 0.03223 | 71.0 | 417 | 0.05281 | 102.0 |
| 400 | 659 | 0.04539 | 66.0 | 413 | 0.05684 | 103.0 |
| 425 | 655 | 0.05213 | 65.0 | 413 | 0.05682 | 103.0 |
| 450 | 659 | 0.05978 | 74.0 | 412 | 0.06564 | 107.0 |
| 475 | 660 | 0.06377 | 87.0 | 410 | 0.06697 | 88.0 |
| 500 | 663 | 0.06403 | 83.0 | 408 | 0.07792 | 71.0 |
| 525 | 659 | 0.06294 | 87.0 | 410 | 0.05465 | 72.0 |
| 550 | 660 | 0.05635 | 81.0 | 411 | 0.04818 | 73.0 |
| 575 | 658 | 0.05481 | 85.0 | 410 | 0.04613 | 65.0 |
| 600 | 658 | 0.05262 | 78.0 | 408 | 0.04116 | 44.0 |
| 625 | 660 | 0.04922 | 72.0 | 406 | 0.03855 | 39.0 |
| 650 | 664 | 0.04025 | 77.0 | 407 | 0.03526 | 46.0 |
| 675 | 662 | 0.04377 | 64.0 | 404 | 0.04254 | 45.0 |
| 700 | 665 | 0.04795 | 52.0 | 406 | 0.03644 | 53.0 |
| 725 | 649 | 0.04026 | 42.0 | 406 | 0.03389 | 49.0 |

Table S4: profile d

| Distance (km) | 660 km discontinuity | Peak amplitude of the 660 km discontinuity | Stacking points | 410 km discontinuity | Peak amplitude of the 410 km discontinuity | Stacking points |
| --- | --- | --- | --- | --- | --- | --- |
| -725 | 636 | 0.03395 | 16.0 |  |  |  |
| -700 | 677 | 0.02955 | 18.0 |  |  |  |
| -675 | 685 | 0.04273 | 19.0 |  |  |  |
| -650 | 680 | 0.03995 | 18.0 |  |  |  |
| -625 | 678 | 0.04970 | 22.0 |  |  |  |
| -600 | 679 | 0.03599 | 25.0 |  |  |  |
| -575 | 682 | 0.04691 | 27.0 |  |  |  |
| -550 | 678 | 0.04254 | 31.0 |  |  |  |
| -525 | 680 | 0.03130 | 35.0 |  |  |  |
| -500 | 646 | 0.02719 | 27.0 |  |  |  |
| -475 | 624 | 0.03096 | 21.0 |  |  |  |
| -450 | 632 | 0.03523 | 18.0 |  |  |  |
| -425 | 628 | 0.05321 | 11.0 |  |  |  |
| -400 | 629 | 0.04628 | 12.0 |  |  |  |
| -375 | 681 | 0.03215 | 12.0 |  |  |  |
| -350 | 617 | 0.02204 | 27.0 |  |  |  |
| -325 | 699 | 0.01789 | 18.0 |  |  |  |
| -300 | 694 | 0.03537 | 23.0 |  |  |  |
| -275 | 690 | 0.02698 | 34.0 |  |  |  |
| -250 | 680 | 0.02225 | 43.0 |  |  |  |
| -225 | 674 | 0.02978 | 47.0 |  |  |  |
| -200 | 683 | 0.02047 | 61.0 |  |  |  |
| -175 | 699 | 0.01993 | 80.0 |  |  |  |
| -150 | 675 | 0.02075 | 100.0 |  |  |  |
| -125 | 670 | 0.02052 | 97.0 |  |  |  |
| -100 | 666 | 0.02250 | 89.0 |  |  |  |
| -75 | 667 | 0.01906 | 113.0 |  |  |  |
| -50 | 665 | 0.01887 | 118.0 |  |  |  |
| -25 | 667 | 0.02047 | 124.0 |  |  |  |
| 0 | 675 | 0.02211 | 135.0 | 428 | 0.02292 | 158.0 |
| 25 | 657 | 0.01873 | 170.0 | 423 | 0.02611 | 163.0 |
| 50 | 657 | 0.02343 | 184.0 | 417 | 0.02688 | 177.0 |
| 75 | 654 | 0.02032 | 181.0 | 424 | 0.02752 | 168.0 |
| 100 | 650 | 0.01776 | 164.0 | 420 | 0.02273 | 169.0 |
| 125 | 649 | 0.01612 | 152.0 | 425 | 0.02446 | 133.0 |
| 150 | 668 | 0.01491 | 156.0 | 428 | 0.02038 | 111.0 |
| 175 | 640 | 0.02137 | 143.0 | 418 | 0.02666 | 107.0 |
| 200 | 637 | 0.02002 | 117.0 | 419 | 0.03701 | 92.0 |
| 225 | 666 | 0.01661 | 105.0 | 419 | 0.04035 | 77.0 |
| 250 | 663 | 0.01910 | 94.0 | 420 | 0.03757 | 75.0 |
| 275 | 664 | 0.02602 | 91.0 | 369 | 0.03102 | 35.0 |
| 300 | 659 | 0.02795 | 80.0 | 412 | 0.02678 | 30.0 |
| 325 | 655 | 0.03129 | 70.0 | 416 | 0.03673 | 35.0 |
| 350 | 656 | 0.03474 | 55.0 | 414 | 0.04247 | 50.0 |
| 375 | 653 | 0.03272 | 47.0 | 411 | 0.04983 | 58.0 |
| 400 | 656 | 0.03643 | 56.0 | 409 | 0.05215 | 58.0 |
| 425 | 657 | 0.05120 | 61.0 | 407 | 0.06427 | 41.0 |
| 450 | 654 | 0.05194 | 69.0 | 406 | 0.05723 | 38.0 |
| 475 | 655 | 0.05106 | 74.0 | 407 | 0.06184 | 35.0 |
| 500 | 654 | 0.04351 | 83.0 | 408 | 0.05613 | 30.0 |
| 525 | 655 | 0.03002 | 80.0 | 407 | 0.03689 | 22.0 |
| 550 | 653 | 0.02640 | 67.0 | 420 | 0.02680 | 20.0 |
| 575 | 652 | 0.02868 | 47.0 | 416 | 0.02771 | 24.0 |
| 600 | 659 | 0.02522 | 40.0 | 409 | 0.01978 | 23.0 |
| 625 | 655 | 0.02787 | 42.0 | 409 | 0.01626 | 27.0 |
| 650 | 654 | 0.02149 | 41.0 | 409 | 0.01481 | 31.0 |
| 675 | 652 | 0.02880 | 30.0 | 408 | 0.02723 | 37.0 |
| 700 | 659 | 0.02323 | 30.0 | 414 | 0.03563 | 41.0 |
| 725 | 659 | 0.01816 | 36.0 | 417 | 0.03354 | 43.0 |
